# Kentucky State Dental Association

**Published:** 1875-11

**Authors:** A. O. Rawls, Chas. E. Dunn


### Proceedings of Societies.

### KENTUCKY STATE DENTAL ASSOCIATION.

Fifth annual meeting of the Kentucky State Dental Association convened in the Parlors of the Phœnix Hotel, Lexington, June 1st, 1875.

The Association was called to order by the President, Dr. B. Oscar Doyle. The Rev. Mr. Christie being introduced; opened the meeting by prayer, after which the roll was called and the following members answered:

B. Oscar Doyle, President.
A. W. Smith, Vice-President.
C. E. Dunn, Secretary.
J. F. Canine, Treasurer.

F. Peabody, W. G. Redman and A. O. Rawls, Board of Censors.

J. F. Canine, W. G. Redman, Executive Committee.

Drs. S. Driggs, W. N. Goddard, J. W. Grant, G. W. Priest, J. O. Dedman, W. Van Antwerp.

Visitors present:

A. S. Talbert, J. S. McMillan, P. L. Dedman, S. P. Grant, J. Hoopper, R. Peckover, W. D. Allen, N. A. Beemer, W. S. Moores, D. A. Morse, J. B. Kidd, H. J. Billings.

President appointed Dr. Rawls on Executive Committee, pro tem in place of Dr. Rogers, absent.

On motion of Dr. Goddard, all dentists and physicians of Lexington and vicinity, were invited to attend and participate in the discussions.

The Executive Committee reported subjects for discussion as follows:

1st. Prevention and treatment of caries upon the proximate surfaces of teeth, and is it good practice to extract teeth from crowded dentures to prevent or arrest decay.

2d. Filling teeth; comparative value of different materials; description of methods of operating.

3d. Probable causes of frequent failures of fillings in the proximate surfaces of molars and bicuspids.

4th. Treatment of dental pulps for their preservation.

5th. Mechanical dentistry. The relative value of celluloid and rubber as a base for artificial teeth.

Dr. Goddard exhibited some very peculiar specimens, consisting of some dozen perfectly formed teeth taken from the socket of a deciduous canine tooth, by Dr. D. N. Morse, of Richmond. None of the teeth being larger than small grains of wheat. On motion dues of Dr. W. H. Shadoan were remitted and his name dropped from the roll at his request.

Adjourned, to meet at 8 o clock p. m.

EVENING SESSION.

Association called to order by the President. After some consideration of the constitution and by-laws, the first subject on programme for discussion, that of prevention and treatment of caries upon the proximate surface of the teeth and is it good practice to extract teeth from crowded dentures to prevent or arrest decay, was taken up and discussed by Drs. Canine, Redman, Van Antwerp, Rawls, Peabody, Goddard, Priest, Smith and Doyle, when on motion futher discussion was postponed and meeting adjourned to meet at 8:30 next morning.

### SECOND DAY.

Association called to order by President Doyle. Dr. Lambkin, member from Georgetown, was reported present, and the following visitors: Dentists, Drs. Kelley and Justice. Physicians, Drs. Buckner and Chinn. The President announced next business in order, further discussion of first subject, which was continued for short time and passed. The second subject, Filling teeth; comparative value of different materials; description of methods of operating, was taken up and in connection with which Dr. Rawls read an essay on the following subject. Does past experience justify the use of cohesive foil for filling teeth.

### DISCUSSION.

Dr. Peabody: Our profession is a progressive one. In order to progress we sometimes have to retrograde. While I was at one time an extremest in the use of soft or non-cohesive foil, and afterwards leaned towards the exclusive use of cohesive foil, I am now thoroughly eclectic in everything. I do not believe that any rigid rule from which there shall be no deviation in regard to the use of materials can be laid down in the practice of our profession. I find good in all, what I find to be good I adopt, that which is not good I discard. I can not agree with the position taken by those who use cohesive foil exclusively, yet I think cohesive foil, at times, necessary in the performance of a certain class of operations which can not be accomplished with soft foil. For general use I should certainly give my preference to non-cohesive foil. Gold introduced on the wedge principle, will give better results and save more teeth when used by the average dentist than gold which depends on the welding prin ciple for its retention. Cohesive foil, if not placed just where it is wanted, can never be made to go there, the force of an instrument on it tends more to harden its surface than to force it to any desired position, and the more force there is applied the harder its surface becomes. Soft foil, on the contrary, can be forced to its position, if not placed there by the foil carriers. The force of an instrument on soft foil is felt all through it, and every blow of the mallet and every ounce of pressure brings the foil more closely in contact with the walls of the cavity. I find many carefully made, handsome cohesive foil fillings in approximal cavities that fail at the cervical wall. That point, being constantly bathed by the mucus of the gums, is in more danger than any other portion of the filling, therefore more care is required, more thorough adjustment of the foil, that it may be brought into the closest possible contact with the dentine and enamel, this can more readily be done with soft than cohesive foil, particularly in those cavities where the eye can not rest on all its parts. In compound cavities it has for years been my practice to put one or more large cylinders of soft foil at the cervical portion, for a while I was content with this, but I gradually added more soft foil, using less cohesive, until I now fill three-fourths or more of the cavity with soft foil, bringing it in contact with all the edges, using cohesive foil merely as a key or capping for the coronal portion. In cylinder fillings of soft foil in crown cavities I often introduce cohesive foil in the center, to act as a key and to give it additional density. I think the idea that the harder a filling is, the better it is, is a mistaken one. We see gold fillings of soft foil, put in twenty and even thirty years in the crowns of molar teeth, by hand pressure, before the use of the mallet was known, that have saved the teeth perfectly and have worn away little more than the tooth has. What more is desired?

Certainly careless and hurried operations will come nearer succeeding with soft foil than with cohesive. Where soft foil can be used equally well with cohesive, less time is required to make a good gold filling than with cohesive foil, thereby saving our own strength and that of our patients. I believe the best gold filling that can be made, is one where soft foil is in contact with the walls and cohesive in the center. Our aim should be to perfect ourselves in the use of both, so we may handle both with equal skill.

Dr. A. W. Smith: I am not an extremest in the use of cohesive foil, although I give it greatly the preference in my practice generally. I use some soft foil in connection with the cohesive and also make a few soft foil fillings but only in cases of cavities with projected walls, such as a cavity in the grinding surface of molars. I believe in eclecticism, in the true sense of the term, in the practice of dentistry; take the best of all that is good in all cases, use the screws to retain fillings when practicable, I use the heavy foils generally, Nos. 20, 40 and 60, seldom as high as No. 120. Think that we generally mallet fillings too much. Fractures of the enamel so often spoken of and urged as an objection to the mallet, are caused by the careless use of the mallet. Any operator that thoroughly understands the use of cohesive foil and with an ordinary amount of caution, need not commit this error.

I seldom have trouble at the cervical wall; use the rubber dam exclusively; use Varney's pluggers or some of similar pattern; separate teeth with pledgets of cotton; prefer contour fillings for bicuspids. I should not cut between teeth simply to find out if the tooth was decayed, would consider it mal practice, have very little use for the file or disk except to finish fillings.

I will say a word in regard to one class of fillings and one kind of material.

Most of my patients are children and I take great pleasure in trying to save their first permanent molars, which I regard as the most important in the set. These teeth generally decay very early, and often before the patient is eight years old, it will require from fifteen to twenty-five grains of gold to fill them. To prepare these cavities, the patient need not be confined in the same position more than two or three minutes at a time, and in no case will it require more than five minutes to introduce the gold. After being introduced it can be condensed slowly without wearying the patient. I use non-adhesive gold in the form of cylinders. No cylinder to contain more than one and a half grains of gold. No napkins, rubber dams or other disagreeable appliances are necessary for keeping out the moisture. Fillings, properly made in this way, will never fail, and can be put in, in less than half the time than by any other method. The object in making those and all other fillings should be to accomplish the desired end in the shortest time and without the least annoyance and expense possible to the patient. I believe that soft gold in the shape of cylinders, properly handled will save more teeth than gold in any other form or any other material that has been used for that purpose.

Further discussion postponed until evening.

Dr. Priest offered the following resolution:

*Resolved*, That a clinic be held this afternoon at 2 o'clock, at the offices of Drs. Drigg's and Rawls, and that as many operators be appointed as can be supplied with chairs, it being the desire of many dentists present to see the different modes of filling teeth with both soft and cohesive foil.

On motion, Drs. Rawls and Smith were appointed to operate with cohesive foil, and Drs. Redman and Doyle, to operate with soft foil.

On motion of Dr. Goddard, the proceedings of this meeting were ordered to be printed in the DENTAL REGISTER and the editor requested to furnish each member with a copy of the same, and the bill ordered paid by the Treasurer on order of the President. On motion, adjourned, to meet at 8 o'clock p. m.

### EVENING SESSION.

Association called to order by the President, who announced that the next business in order would be continuation of discussion on the subject of Comparative value of materials for filling teeth, description of methods of operating.

Dr. Smith wanted to know Dr. Redman's objections to amalgam.

Dr. Redman: I have no use for it, it may be good in its place, but I do not think that is in the teeth.

Dr. Morse: I would like to hear this subject freely discussed, I have never used it in my life and have no use for it at all, but if any one can say anything in its favor, I would like to hear it.

Dr. Goddard said he had used amalgam and had seen a tooth lately, which he filled with amalgam twenty-five years ago, and which, at the time it was done, the patient wished to have extracted instead of filled, thought that amalgam should be used dry or free as possible from mercury, with as careful preparation of the cavity as though gold was to be used. Dr. Grant said that he felt a great interest in the subject, for a long time he did not use amalgam but now used it in some cases, but generally preferred tin for cheap fillings.

Dr. Van Antwerp: I have used amalgam in my practice but the longer I use it the less use I have for it, however I think in a few cases it might be used successfully; very much depends on the quality of the amalgam as well as skill of the manipulator; have seen tin fillings which had been in forty-three years, and much prefer to use tin as a cheap filling and amalgam little as possible.

Dr. Smith used amalgam sometimes, but had little use for it. In large cavities where pulp was dead generally filled roots with Guilloi's cement and over the same with gold. Had watched some of the cases for four or five years and find they are doing well.

Dr. Redman had tried these plans for ten years and thought that pecking on cohesive gold filling with the mallet could not be done without injury to the tooth, but with soft foil thought the most perfect filling could be produced. He objected to amalgam, many years ago had seen a lady with beautiful teeth, but some of her back teeth were filled with amalgam, and after suffering a long time with neuralgia she finally lost them entirely.

Dr. Goddard: The amalgam made use of at the time Dr. R. speaks of, was very different from the amalgam made now. Many years ago I had a tin filling in my tooth which

I removed, and went to Dr. —— to have it filled with his new plastic filling, on my way stopped and bought some cake, eating along on the way to his office, went in and had the tooth filled and when I came out took my knife and dug the filling out and when I did so, also dug some of the cake from the cavity, now we all know that a tooth filled in that manner can not stand. Amalgam, in those days was made by filing old silver dollars and rubbing the filings up with mercury.

Dr. Driggs: I would like to hear from any one who has noticed any galvanic action where gold and amalgam have been used in the same mouth.

Dr. Van Antwerp answered that he had noticed cases of galvanic action where certain conditions favored the conduction of galvanic currents. Believed that action of this character takes place in mouths where gold and amalgam had been used for filling the teeth, but thought that certain other conditions were necessary to cause galvanic action besides the mere fact of both materials, being in the same mouth.

Dr. Rawls: I have seen very many teeth saved by filling with amalgam which I had reason to believe would have been sacrificed in a short time, had they been filled with other material, and though I do not use it often yet I think there are cases in which amalgam can be used with as good, if not better results than any cheap material for filling teeth, and to better advantage, in a few cases, than common gold.

This morning Dr. Redman, in his remarks on the subject, contended that soft foil could be used for filling in the six year molars with better success than any other material. Now this may do providing the cavities are not extensive and the tooth structure ordinarily good, but when the cavities are very large, occupying most of the grinding surface and broken through into the approximal and the tooth of indifferent structure, then I would not consider it good practice to fill entirely with gold. In most cases when the six year molars of children are badly decayed, and when they can be filled, I believe it better practice to coat the walls of the cavity with a thin varnish or solution of gum shellac, fill the greater portion of the cavity with os-artificial, Guilloi's cement or some kindred preparation and complete the operation by filling over with amalgam. Teeth thus filled will last until the patient is, say twenty years of age, when the tooth structure will be better and, then if desired, the amalgam can be removed and replaced with gold.

It is true that by filing or chiseling away a goodly amount of tooth structure in these cases we could fill at once with soft foil, and say of the fillings they were good, that is, in themselves, but is this correct practice? Is it right to sacrifice so much tooth structure and thereby prevent natural antagonism of the tooth with its fellow, when by other means, such results could have been avoided. Many of the bad results following the use of amalgam are caused by bad manipulation, men sometimes take for granted that because amalgam is a cheap filling it should be put in the cavity in the cheapest way, when really if any difference be made at all in the preparation of the cavity, it should be made in favor of amalgam. It was said this morning by Dr. Peabody, that when using cohesive gold for filling approximal cavities he invariably noticed failure of the fillings at the cervical wall. I desire now to know of the gentleman, if there exists any good reason for failure at this particular part of the filling. Surely the structure of the gold has nothing to do with it, neither can it be proven that any peculiar chemical agent is used in its manufacture which might by any means bring about such results. These things being out of the question, we can only attribute the failures to a misapplication of the material. A moment's thought should convince any one that the gold could be placed as completely in contact with the cervical wall of a cavity as with any other wall of the same. It has been said, by Dr. Priest I believe, that in drilling retaining points for this class of work, the drill was apt to plunge into the pulp cavity, or so nearly to it that in filling, the thin intervening dentine would be driven in upon the pulp and cause inflammation and final loss of the tooth. This is certainly a very weak argument against the use of cohesive foil. Must we be governed in our practice by the mistakes of men? Must we condemn the use of cohesive foil, for the reason that bungling hands occasionally misdirect a drill. One other point in connection with this subject has not been touched. Suppose a case is presented of an incisor badly decayed on the approximaal surface, the pulp exposed and healthy, the palatine and labial plates of enamel broken down to a level with exposed portion of the pulp; you desire to protect the pulp by capping and then fill over the capping. Now tell me how you are to proceed with non-cohesive gold, where are your walls within which to confine your cylinders. There are also other cases in which no matter how you manipulate non-cohesive foil, it can not be made to serve the purpose.

I do not wish however to be understood as condemning the use of any material for filling teeth which has thus far proved of value, but on the contrary think it the duty of every man in the profession, who has its interests at heart, to thoroughly acquire the use of all materials, and acquaint themselves with all modes of operating whereby the most good can be accomplished.

Dr. Doyle: I would state in reference to this subject, that during my studies as a student, I never saw an amalgam filling, and had been in practice some time before I used it. A gentleman called on me whose back teeth had been filled with amalgam and his front teeth with gold. The gold fillings having failed he had them refilled with gold and they failed the second time, and, since the amalgam fillings still remained good, he concluded to have that material used in refilling his front teeth and came to me for that purpose. I remember well the first tooth I filled with amalgam, that tooth still remains good. I do not think that amalgam can be used successfully in ordinary sized cavities, and have never seen amalgam successful where the walls were thin and light, though I think it has stood the longest in teeth when almost any material could have been used. I believe that galvanic action is some times occasioned when gold and amalgam are used in the same mouth. In regard to other materials for filling teeth, I must say that I honor and respect any member of the profession who strives to succeed, whether he uses cohesive, non-cohesive, amalgam or tin. I have endeavored to study thoroughly, all the various methods of filling teeth, and think at one time I ran into excess in the use of cohesive gold. It was a very seductive practice and I was somewhat deceived.

The Board of Censors reported they had received applications for membership from the following persons:

W. S. Moores, D. D. S., M. D., Maysville, Ky.; J. Hooper, of Owington; W. W. Justice, of Winchester; J. N. Floore, Frankfort; J. T. McMillan, Paris; T. D. Kelley, Lexington; N. J. Billings, M. D., Louisville; and cheerfully recommended them to the Association. The report was received and ballot ordered separately and each one having received a majority of all votes cast were severally declared duly elected.

On motion, subject No. 3 was passed. On motion, of Dr. Rawls, the election of officers and selection of place for next meeting was made the special order of business for Thursday morning at eleven o'clock. Adjourned to 8½ o'clock Thursday morning.

### THIRD DAY—MORNING SESSION.

Association called to order at 9 o'clock. Treatment of exposed pulps for their preservation was taken up.

Dr. Van Antwerp: I have never used lacto-phosphate of lime in the treatment of exposed pulps, but have used pepsin when the pulp was partially dead and in three I know I have succeeded in saving a portion of the pulp alive. The pepsin produced some little irritation and in order to subdue the same and prevent inflammation, I used iodine or carbolic acid. The teeth are in apparent good condition to-day.

Dr. Smith: My experience with lacto-phosphate of lime is small. Have not had as good chances for its use probably, as others, but so far my preference is for pepsin. When I get the pulp and cavity prepared I use Guilloi's cement with creosote and then fill over with the cement prepared in the usual way and afterwards fill with gold.

Dr. Rawls: This subject is of very great interest to me, I have made it a special study for several years. Did not hear all that had been said on the subject by previous speakers, but judging from the tone of remarks made in my hearing I think there must be some mistake in reference to the properties and intended use of lacto-phosphate of lime and pepsin. Lacto-phosphate of lime is not intended, as might be inferred from what has been said, as an agent to destroy or dissolve albumen as does pepsin. It was first introduced to notice of the profession by Dr. Cravens, of Kansas City, and at the time was intended to be used over an exposed, healthy pulp, in order that it might in some way cause deposition of dentine over the point of exposure, while pepsin is intended to be used as a cleansing agent, and especially to remove or dissolve albumen from dead portions of the pulp or from the pulp cavity and canals when pulp and nerve are entirely dead.

It matters not what we use as a capping for exposed pulps providing the latter be healthy and the substance used for capping be indestructible in its position, will conform itself to the shape of the parts without undue pressure on the pulp, will not be irritating and will harden sufficiently to admit of filling over with gold without being broken or displaced. We have not as yet a material combining all these requisites, but the oxy-chloride of zinc, Guilloi's cement and similar preparations can be used successfully by intervening a substance between the capping and pulp, to prevent irritating effects of chloride of zinc, which is used in all of these preparations. In my practice I not only endeavor to save pulps alive when intact, but also save the remaining nerve branches when the pulp is dead to opening into the root or roots, think the latter, when it is practicable, much better than to destroy the pulp to the apex of the root.

Dr. Redman: I believe in destroying the nerves to apex of root, think this practice spoken of by Dr. Rawls is fancy and not to be relied on, is unpracticable in the majority of cases.

Dr. Priest: I am of the opinion that we should save all the life of a tooth possible and think that part of a live nerve is to be preferred to no nerve at all, however I think Dr. Rawls has made statements hard to carry out in general practice.

Dr. Peabody: I have just come in and have heard only a portion of the remarks on this subject, think those made by Dr. Rawls cover the ground, as taught by professors of the day. The remarks of Dr. Redman are also in part correct, some years ago I made remarks to the effect that I did not consider it necessary for a surgeon to use barbed instruments for the removal of any vital part. I practice that, though I do not believe in it. I usually resort to every means possible, to save the nerve alive and when I have to destroy it I use tannic acid, introduce it into the cavity, allowing it to remain several days and find after one or two applications of the tannic I can remove the pulp or nerve branches completely, the tannic acid having turned them into leather.

The Board of Censors made a report recommending D. T Morse, of Richmond, for membership. On motion, report received and ballot ordered, which resulted in his election.

The Executive Committee reported they had examined the Treasurer's report and found the same correct, with balance of fifty-one dollars and fifty cents in the treasury at commencement of session.

The President announced the next business in order was election of officers and selection of place of meeting for June, 1876.

The Association proceeded to election of officers with the following results:

For President, Charles E. Dunn, Louisville.
" Vice President, W. S. Moores, Maysville,
" Secretary, A. O. Rawls, Lexington.
" Treasurer, J. F. Canine, Louisville.
" Censor, S. Driggs, Lexington.
" Executive Committee, W. N. Goddard, Louisville.

Drs. Smith, Rawls, Moores, McMillan and Billings were elected delegates to American Dental Association, with S. Driggs, W. G. Redman, W. W. Justice, J. H. Floore and J.

Hooper, alternates, also Dr. Peabody, delegate to Southern Dental Association, Dr. Driggs, alternate.

Louisville, Frankford, Mammoth Cave and Covington, were nominated as places for next meeting, Mammoth Cave having received a majority of all votes cast was declared the place for holding the meeting in June, 1876.

The officers elect for ensuing year were introduced and the retiring President, Dr. B. Oscar Doyle, then read an address in which very much of interest to the profession was presented. Adjourned to 8 o'clock p. m.

### NIGHT SESSION.

The Association was called to order by the President, Chas. E. Dunn. On motion, the fourth subject passed and the next one in order, that of Mechanical Dentistry, relative value of rubber and celluloid was taken up.

Dr. Doyle said that very much improvement had been made in the manufacture of celluloid plates and that as now used it required much less pressure to close the flasks than formerly. Believed it much better than rubber.

Dr. G. W. Redman spoke in favor of the dry or Finley Hunt apparatus, said he had tried steam and oil but would give preference to the Hunt process. Subject passed and that of volunteer essays taken up.

Dr. F. Peabody favored the Association with a paper on alveolar abscess, one of the salient points of which was the merits of lead wire as an application for filling roots of teeth where there existed a peculiar form of chronic abscess. On motion the vote declaring Mammoth Cave as place of next meeting was reconsidered and Louisville selected instead. Adjourned to meet in the city of Louisville first Tuesday in June, 1876.

A. O. RAWLS, Secretary. CHAS. E. DUNN, President.

